# A Blockchain and Artificial Intelligence-Based, Patient-Centric Healthcare System for Combating the COVID-19 Pandemic: Opportunities and Applications

**DOI:** 10.3390/healthcare9081019

**Published:** 2021-08-08

**Authors:** Mohamed Yaseen Jabarulla, Heung-No Lee

**Affiliations:** School of Electrical Engineering and Computer Science, Gwangju Institute of Science and Technology, Gwangju 61005, Korea; yaseen@gm.gist.ac.kr

**Keywords:** digital healthcare, patient-centric, blockchain, artificial intelligence, federated learning, coronavirus (COVID-19), pandemic management, healthcare transformation, public health strategies

## Abstract

The world is facing multiple healthcare challenges because of the emergence of the COVID-19 (coronavirus) pandemic. The pandemic has exposed the limitations of handling public healthcare emergencies using existing digital healthcare technologies. Thus, the COVID-19 situation has forced research institutes and countries to rethink healthcare delivery solutions to ensure continuity of services while people stay at home and practice social distancing. Recently, several researchers have focused on disruptive technologies, such as blockchain and artificial intelligence (AI), to improve the digital healthcare workflow during COVID-19. Blockchain could combat pandemics by enabling decentralized healthcare data sharing, protecting users’ privacy, providing data empowerment, and ensuring reliable data management during outbreak tracking. In addition, AI provides intelligent computer-aided solutions by analyzing a patient’s medical images and symptoms caused by coronavirus for efficient treatments, future outbreak prediction, and drug manufacturing. Integrating both blockchain and AI could transform the existing healthcare ecosystem by democratizing and optimizing clinical workflows. In this article, we begin with an overview of digital healthcare services and problems that have arisen during the COVID-19 pandemic. Next, we conceptually propose a decentralized, patient-centric healthcare framework based on blockchain and AI to mitigate COVID-19 challenges. Then, we explore the significant applications of integrated blockchain and AI technologies to augment existing public healthcare strategies for tackling COVID-19. Finally, we highlight the challenges and implications for future research within a patient-centric paradigm.

## 1. Introduction

The novel coronavirus disease (COVID-19) has spread to almost every country since the outbreak in December 2019 from Wuhan, China. The severity of this epidemic became extensive within a month of the virus’s widescale spread. Thus, a Public Health Emergency of International Concern (PHEIC) was declared by the World Health Organization (WHO) [1]. The outbreak forced several nations to close their borders, maintain lockdowns, and practice social distancing to limit the spread of COVID-19. These led to massive interruptions in the economy of many sectors, such as industry, insurance, agriculture, supply chains, transport, and tourism [2]. The pandemic has had an unexpected impact at the global level, not just on an economic scale, but also pushing healthcare systems around the world to their limits, such as through a lack of personal protective equipment (PPE) for healthcare workers and by causing difficulties in diagnosing and monitoring large populations [3]. In general, the healthcare system has operated in a closed ecosystem of siloed institutions, where healthcare professionals (i.e., doctors, radiologists, clinicians, and researchers) have served as the primary stakeholders of medical information. The flow of information has gone in one direction, i.e., healthcare expert to patient. However, in the era of digitized patient health records, data are growing and flowing across a closed healthcare system faster than ever before. The one-to-one flow of information is giving way to a multiplicity of information, sharing relationships with many-to-many, one-to-many, and many-to-one [4]. In such cases, most coronavirus information collected from the public, hospitals, and clinical laboratories may not be faithful, since the data are not gathered according to set guidelines [5] and are not monitored or stored appropriately because of the vastness of digitized patient health records. The existing healthcare technology requires trustable data, which is crucial to providing the correct widespread information about the novel coronavirus. Furthermore, the virus test procedure using medical tools for detecting coronavirus infections often takes several days to complete because of the inaccuracy and manual processing of large volumes of data. Finally, tracking or surveilling infected patients or their contacts raises several privacy issues [6]. These insufficiencies exposed by COVID-19 have prompted healthcare organizations to transform the existing digital healthcare system to combat pandemic situations. Overall, the digital healthcare ecosystem needs to facilitate clinical trials, frontline care, data surveillance, medical billing, telemedicine, drug delivery, treatment facilities, and strategy discovery. In addition, it is essential to design a more patient-centric and democratized digital healthcare ecosystem for combating COVID-19 and future pandemics by using digital platforms.

Recently, several researchers have focused on utilizing disruptive technologies, such as blockchain and artificial intelligence (AI), to provide solutions for these ongoing COVID-19 crises [7,8]. Blockchain is a peer-to-peer (P2P) distributed and shared ledger, where transactions are digitally recorded into blocks. The nodes (miners) of the blockchain network are responsible for linking the blocks to each other in chronological order. Blockchain nodes contain a copy of the stored information and keep their network active [9]. Thus, blockchain provides the entire history or provenance of data. It is possible to store sample test results, patient records, discharge summaries, and vaccination statuses in a blockchain digital ledger. These will support clinical laboratories, patients, hospitals, and government-funded healthcare organizations in a decentralized way to manage healthcare information using self-executing contracts called “smart contracts” [8]. Smart contracts are computer programs that execute the predefined terms of an agreement between participants when certain conditions are met within the blockchain network [10]. Furthermore, smart contracts based on blockchain technology could automate auditing processes, medical supply chain management, outbreak tracking, and remote patient monitoring [10]. On the other hand, AI technologies, such as machine learning and deep learning, have been used as powerful tools for enhancing COVID-19 detection, diagnosis, and vaccination/drug discovery, and for performing extensive data analysis [11]. In addition, the federated learning paradigm [12,13] has gained traction for healthcare applications to solve the data privacy and governance problems by training AI models collaboratively without sharing the raw datasets. Thus, AI could process an enormous amount of data in less time and at a fraction of the cost by performing tasks that are difficult to achieve manually. Meanwhile, the blockchain could promote secure data access and interoperability while protecting the privacy and security of health data [14]. Integrated blockchain and AI technology could reshape the healthcare ecosystem by advancing the patient-centric approach [15,16,17]. A patient-centric approach could provide a viable solution to cope with the coronavirus epidemic for disseminating treatment and managing pandemic situations.

Although researchers have reviewed blockchain and AI to combat COVID-19 [18], these reviews mainly focused on the role of blockchain, such as the development of data storage, managing big data, and security issues for COVID-19 patients [14]. Other reviews focused on analytics and decision tools for healthcare professionals to combat COVID-19 using AI technologies [11]. However, these reviews lacked a concrete and comprehensive study on integrating blockchain and AI for COVID-19 responses based on a patient-centric approach in the healthcare ecosystem, a limitation that was the primary driver for conducting our research. This paper aimed to provide and explore insight into combined blockchain and AI technology to mitigate the COVID-19 pandemic’s challenges by transforming the traditional healthcare ecosystem. Then, we discuss the services and practical applications of using these innovative technologies to facilitate COVID-19 healthcare strategies. The contributions of this article are as follows:

1. We conceptually redefined the traditional healthcare model by integrating blockchain and AI for tackling COVID-19 in a patient-centric paradigm.
2. We exploited the existing public health strategies, such as patient information sharing, data management for diagnosing the infection, contact tracing, monitoring, and mitigation of the impact on healthcare, using the proposed decentralized, patient-centric frameworks.
3. Based on the study, we discussed the challenges, solutions, and future research directions that are anticipated to be of significant value for patients and healthcare organizations.

This work is organized as follows: Section 2 includes related research work and recent trends in healthcare systems that utilize blockchain and AI technologies. Section 3 provides an overview of digital healthcare services and describes the background of blockchain and AI technologies. In Section 4, we exploit a blockchain- and AI-based conceptual framework for delivering patient-centric healthcare services to combat COVID-19. Section 5 explores the potential applications of the decentralized, patient-centric framework to facilitate pandemic healthcare strategies. In Section 6, we discuss the relevant open issues, possible solutions, and further research directions. Finally, we conclude the paper in Section 7.

## 2. Related work

### 2.1. Blockchain and AI in Healthcare Systems

In this section, state-of-the-art research related to healthcare systems based on blockchain and AI is presented. The key risks and issues in a traditional healthcare system include a single point of failure, data alterations, high chances of malicious cyberattacks, centralized authority, high data management cost, and databases that are not transparent. To address these issues, researchers have proposed numerous blockchain-based solutions. The authors of [19] addressed the security and privacy concerns by using a blockchain-based server–client architecture network to store the hashed patients’ data. However, these server–client architectures are prone to a single-point failure. Thus, a blockchain-based distributed mechanism for data accessibility between patients and doctors in a healthcare system is presented in [20]. The authors of [10] designed a smart contract-based, real-time patient monitoring system to record wearable device data as events and share that information with healthcare professionals. The primary goal of this system is to eliminate third parties and resolve the vulnerability issues in remote monitoring. In other studies [21,22], researchers created a secure and trusted digital environment using a smart contract-based healthcare system to prevent data breaches in electronic health records (EHRs). To attain decentralized data management in healthcare, the authors of [16] proposed a framework to store patient EHRs in a decentralized, patient-centric framework that allows patients to control their data using a rule-based smart contract. On the other hand, healthcare systems based on AI [23] require more computational power due to the exponentially increased parameter numbers, complex architectures, and sufficient data to achieve accurate deep learning solutions. Meanwhile, the data are accumulated from different sources and stored on the central server to find a global model. Therefore, researchers have proposed distributed AI approaches based on blockchain that leverages parallel computing power, as well as focuses on distributed data storage. One such effective distributed learning solution is federated learning that trains locally stored data with local computational power while protecting privacy [12]. The federated learning approach allows researchers to obtain decent insight from patient data without revealing any sensitive information (medication history of patients, text messages, and patient names, etc.). Researchers are typically looking for statistical results rather than raw data, and researchers can achieve unbiased statistical insight without even having access to the data itself. Jonathan et al. [24] proposed a conceptual framework based on blockchain-orchestrated federated learning for healthcare consortia. Their architecture provides a privacy-preserving audit trail that logs events in the network without revealing identities. 

### 2.2. Trends in Related Research

More recently, the massive outbreak of the COVID-19 pandemic has prompted various researchers, scientists, and organizations around the world to conduct large-scale research to help develop efficient pandemic management and response strategies. Several patient-centric approaches [15,17] have been studied in the context of the COVID-19 pandemic, such as facilitating pharmaceutical care [25], clinical trials [26], conducting ethical research [27], and health system restructuring [28]. The emergence of disruptive technologies like AI and blockchain leveraged several healthcare applications [11,14,18,29] that emphasized COVID-19 data analysis, data security, data privacy, authenticity, and data sharing at various levels. In this regard, Samuel et al. [30] have reviewed the role of AI in the arena of predicting, contact tracing, forecasting, screening, and drug development for coronavirus and its related epidemic. The authors of [14] reviewed the existing literature on blockchain technology in solving challenging problems due to the COVID-19 pandemic. The authors proposed a blockchain-based platform that discussed significant blockchain applications for solving issues arising from the COVID-19 pandemic. Nguyen et al. [18] introduced a new conceptual architecture that integrates blockchain and AI for combating the COVID-19 pandemic. However, this article only consists of an extensive survey about the latest research efforts on blockchain and AI applications for combating COVID-19. In addition, none of these works provided an overall architecture of the blockchain and AI framework based on a patient-centric paradigm. 

From the abovementioned works, we concluded that, although several studies were focused on the current scenario of the COVID-19 outbreak, they provided only a limited idea about integrating blockchain and AI technologies to combat COVID-19. To the best of our knowledge and at the time of writing, no study has provided a decentralized, patient-centric framework that emphasizes the COVID-19 pandemic and its potential implications using converged blockchain and AI technologies. To this end, our present work has more potential to address the research gaps while presenting the conceptual framework with a detailed explanation of each layer and its functionalities. The purpose of our study was to provide the readers with an initial systematic framework of how integrated blockchain and AI are able to facilitate traditional public healthcare strategies, such as patient information sharing, data management for diagnosing the infection, contact tracing, monitoring, and the mitigation of the impact on healthcare, using the envisioned decentralized, patient-centric frameworks.

## 3. Overview

### 3.1. Digital Healthcare Services during the COVID-19 Pandemic

Digital health can improve pandemic strategies and responses by increasing access to healthcare-related services for individuals and enhancing the experience of delivering or receiving care [31]. Digital health is an umbrella term that includes mobile health (mHealth), electronic health (eHealth), and emerging technologies, such as the use of blockchain, medical internet of things (MIoT), AI, and big data [32,33]. Although some digital technologies, such as telemedicine and telehealth, have existed for decades, they have poor penetration into the healthcare market due to the sparsity of supportive payment structures and heavy regulations [34]. A nationwide surge of COVID-19 cases forced healthcare organizations to transform healthcare delivery by leveraging the power of digital technologies [33,35]. The use of telemedicine for diabetic patients in fighting the COVID-19 pandemic has already been demonstrated [36,37]. Various technologies, such as biosensors, multi-drone systems, and industry 4.0 [29,38,39], could be employed for combating the coronavirus disease. The viewpoint of the authors of [40] represents pandemic management and response strategies based on a methodological application of digital technologies. Their framework highlights the ways in which successful countries (e.g., South Korea, Australia, Germany, Singapore, and Taiwan) have adopted digital technologies in the real world for pandemic healthcare services, such as contact tracing, testing, surveillance, pandemic planning, and quarantine. Table 1 summarizes specific healthcare services [14] by highlighting the functions, challenges, and digital technologies utilized for the pandemic’s management and response strategies.

**Figure 1:**
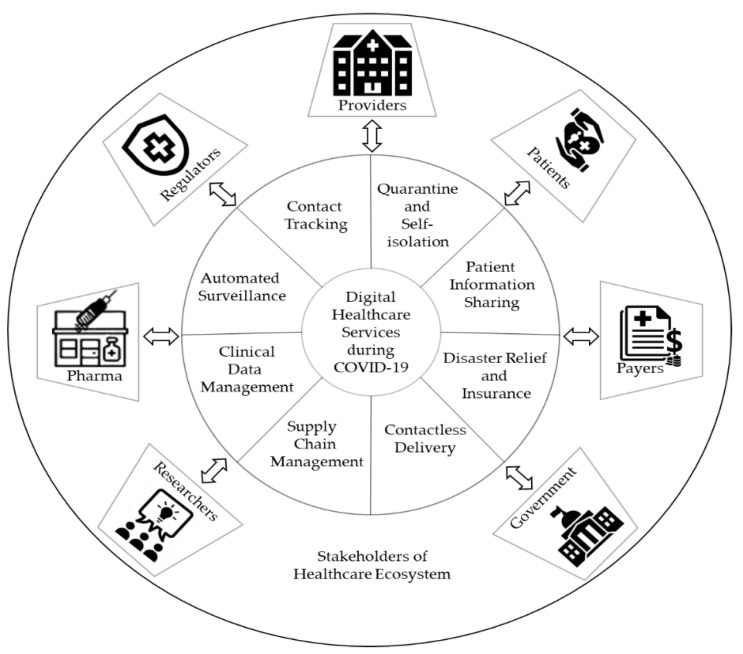
Healthcare ecosystem and digital services for pandemic preparedness and response during COVID-19.

Figure 1 illustrates a representation of a complex healthcare ecosystem with multiple stakeholders who constantly integrate, interrelate, and interoperate with digital technologies during pandemic situations. The key stakeholders of the healthcare ecosystem are described below:

- Patient—anyone who seeks medical care can be termed a patient, and their data play a crucial role in pandemic preparedness and response.
- Providers—Includes physician groups, hospitals, laboratories, doctors, and other healthcare professionals and medical facilities that deliver medical care to patients. Patient data, such as electronic medical records (EMRs), are stored, organized, and managed in a large-scale centralized clinical repository. Providers contribute to clinical teams and researchers by providing health information to combat diseases.
- Payers—A payer is a company (for example, an insurance company) that pays people or bodies, other than the patient, to finance or refund the cost of the medicinal products and healthcare services. A payer is responsible for processing payments, patient eligibility, enrollment, and claims.
- Pharma—Pharmaceutical companies are the makers of vaccines prescribed by healthcare providers. They supply medicines and provide other supporting services, such as patient disease and medication management.
- Researchers—Conduct pharmaceutical and biomedical research. Digital healthcare can augment researchers’ insights by analyzing the healthcare data, clinical trials, and public health research.
- Regulators—Healthcare industries and government agencies that oversee industry standards, enforce and write regulations, and set healthcare policy.
- Government—Handles public safety and emergencies. It implements stay-at-home orders or lockdown to reorganize, rebalance resources, and protect health workers while combating COVID-19. They execute policies that encourage and support innovators to create healthcare solutions based on information technologies where information flows securely to the required parties. Involved in the management of the procurement of PPE kits, medicinal supplies, and appliances/oxygen condensers. Provides staff training on COVID-19 prevention and provision of patient counseling on medicines.

Health services need trustable data to provide the correct information about the novel coronavirus’s spread or outbreak. Multiple efforts are being made around the world to cultivate a patient-centric culture by using ever-growing volumes of research, patient data, and applications of digital technologies [4,42,43,44]. Specifically, disruptive technologies, such as AI and blockchain, are emerging in digital healthcare, which uses the greater availability of health data to identify high-risk patients, track the spread of COVID-19, predict mortality risk, manage healthcare data, and fight against coronavirus and other pandemics [11,14,18]. AI gives us the unprecedented capability to decouple complex variables and reach a nuanced understanding of the effect and cause at the population as well as the individual levels. AI allows organizations to understand what their data are depicting and use that to develop targeted interventions. Blockchain plays a vital role in health information exchange by facilitating the healthcare transition to patient-driven and patient-mediated interoperability [45]. In this article, we propose a decentralized, patient-centric framework that integrates AI and blockchain technologies for tackling COVID-19. We explore the potential applications and use cases of these combined technologies for facilitating the healthcare response to the COVID-19 pandemic [16,41].

### 3.2. Blockchain and Artificial Intelligence Technologies

Blockchain is a promising and revolutionary technology, mainly used where centralization is unnatural and privacy is essential [46,47]. Blockchain [8,48] has a particular interest in health data, with an emphasis on sharing, distribution, and encryption. Decentralization and cryptographic hashing are the fundamental concepts of a blockchain. The contents or databases stored in a blockchain are shared across the network. The network creates a decentralized distributed chain that allows every participant to access the blockchain’s contents. The security of the network is protected by a mechanism called consensus. A consensus mechanism is a fault-tolerant mechanism that uses a set of rules to achieve necessary agreement on the status of the blockchain ledger among all participants. Blockchain consists of three key components: blocks, nodes, and miners. Here, a block is like a record book page that records some or all of the recent transaction data that have not been stored in any prior blocks. Each time a block is completed or mined, it gives way to the next block in the blockchain. Starting from the genesis block, each block consists of data, its own unique nonce, and hash value that links to the previous block via a hash label, which creates a chain of blocks and prevents any modification risks [49]. Nodes are responsible for the functioning of a blockchain network, and ensure the storage of the given data in the distributed ledger. Each node has its copy of the blockchain, and the participating node creates new blocks in the chain for which participants receive a reward. The consensus mechanism provides equal rights to all participants in the network to access the distributed ledger and protect the chain from third-party entities to avoid security issues, such as double-spending attacks [50]. There are several consensus mechanisms available, such as proof-of-work (PoW), Byzantine faulty tolerant (BFT), zero-knowledge proof, and proof-of-stake (PoS) [49]. Furthermore, smart contracts are used to enhance the transparency and trust between two parties by using blockchain technology to enable the creation of accessible and immutable contracts. In a blockchain network, each participant has a unique alphanumeric identification number that shows their transactions. Therefore, every action can be easily monitored and viewed by the participants in the distributed ledger. The smart contract makes secure transactions that help to avoid disruption from centralized authorities. Ethereum Virtual Machine or Solidity platforms [51,52] could be used to build smart contracts for automatizing auditing processes, providing time-bound access to distributed patients’ data, and improving the supply chain management of pharmaceutical products. The healthcare industry has become overloaded by data, and blockchain can provide solutions to healthcare stakeholders to handle this enormous data in reality. In addition, blockchain establishes reliable and privacy-preserving data exchange protocols within the healthcare ecosystem. Blockchain’s immutable and decentralized nature [53] has demonstrated its promising potential in healthcare applications, such as secure data management [54], transparent medical data storage [55], and healthcare data privacy [16,56].

Artificial intelligence (AI) technology appears in every technology field, and is becoming inseparable from daily life activities. Moreover, Accenture researchers [57] predicted that the application of AI in the healthcare market is expected to increase from $600 million to $6.6 billion between the years 2014 and 2021. Recent studies show that AI-based ML and DL models are utilized for solving coronavirus-related issues [11]. ML plays an important role in AI research, and has a huge potential to detect patterns and anomalies of medical image data. Then, it matches those data into learning models to automate decision-making processes for healthcare specialists [58]. For example, ML can be used to perform an automated facial recognition framework to detect temperature on the human body for mitigating coronavirus-infected people [58,59]. Meanwhile, DL models consist of multiple neural network layers to form a deep learning architecture [60]. A deep neural network architecture consists of an input layer, output layer, and a single hidden layer for receiving data samples, training data samples, and generating training outcomes. Here, the depth of the DL architecture depends on the number of hidden layers. Either unsupervised or supervised learning techniques are used to estimate the desired output using unlabeled or labeled data samples, which are associated with the adjustment of the hyper-parameters. Several AI companies have created DL-based models to predict and analyze coronavirus infection [61]. For example, DarwinAI developed a COVID-Net framework using a convolutional neural network architecture to detect COVID-19 from chest radiography images, and Google uses neural network software to predict patient outcomes, such as the length of a visit, odds of death, and readmission possibilities. However, AI algorithms require large, varied, high-quality, and confidential coronavirus datasets that may be siloed across different healthcare institutions. Thus, obtaining patient coronavirus data securely for training the datasets with a global AI model for the detection of positive COVID-19 cases is a challenging task. To address this data security problem, McMahan et al. [12] proposed federated learning frameworks to train an AI model securely by analyzing a broad range of data located at multiple sites. Federated learning secures data and aggregates only the AI model parameters from multiple organizations [13,62,63]. However, several federated learning approaches are based on a centralized server, which raises concerns about the privacy of sensitive data. Thus, researchers proposed blockchain-based federated learning approaches for several applications [63,64,65] to implement asynchronous collaborative AI models between a distributed network. Hence, the blockchain-based federated learning method enables collaborations between several healthcare organizations to train the ML or DL models without relying on any centralized server and avoids the direct sharing of sensitive clinical data with each other. A blockchain smart contract, such as Ethereum, is used to realize the automated management of the entire federal learning method with an incentive mechanism. For instance, Microsoft researchers [66] are developing a system that collaboratively improves ML algorithms hosted on a public blockchain. The collaboration through the system is incentivized, since blockchain makes it possible to reward people who provide data for improving AI models using smart contracts. This decentralized approach to train models preserves privacy and security and ensures the immutability of uploaded AI models via computing and recording their quality in the blockchain. 

**Figure 2:**
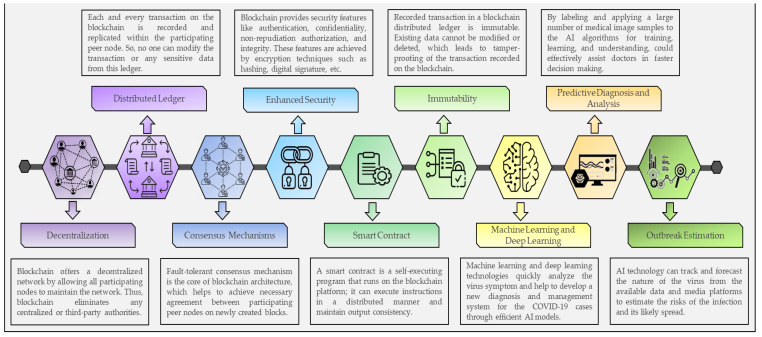
Key features to mitigate COVID-19 challenges using blockchain and AI.

The patient-centric approach aims to facilitate the democratization of ever-growing volumes of patient data and effectively uses it for the application of AI and blockchain to mitigate COVID-19 challenges. Figure 2 depicts the important features of blockchain and AI technology that are essential in combating COVID-19. AI and blockchain are catalyzing the pace of innovation in digital healthcare, which directly impacts patients and service providers. Moreover, these two technologies have their degree of technical complexity as well as ethical concerns, but converging both technologies may be able to redesign the entire traditional healthcare paradigm into a decentralized, patient-centric paradigm to mitigate COVID-19. In summary, the advantages of utilizing blockchain and AI technologies in healthcare to mitigate COVID-19 challenges are as follows:

- Blockchain helps to improve interoperability among different healthcare organizational platforms, such as pharmaceutical needs, hospital databases, supply chain logistics, and insurance claims.
- Storage and management of health record data using blockchain platforms offer patients the protection of their data and provides access to their health records based upon request.
- Blockchain improves information management among stakeholders in the healthcare ecosystem.
- Blockchain reduces centralized control over patient datasets. Thus, it helps to boost medical research and treatment.
- A smart platform can be developed using AI for the automated surveillance, monitoring, detection, and prediction of the spread of this virus.
- The use of AI in reviewing and analyzing radiology images, such as CTs and X-rays, could help to increase COVID-19 detection accuracy.
- AI could automatically estimate the number of positive COVID-19 cases and death cases in any region. In addition, AI helps to determine the most virus-exposed countries, regions, and people to take measures accordingly in advance.
- The application of artificial intelligence (AI) in medication development can help pharmaceutical companies streamline drug repurposing and discovery.

## 4. The Proposed Patient-Centric Framework

**Figure 3:**
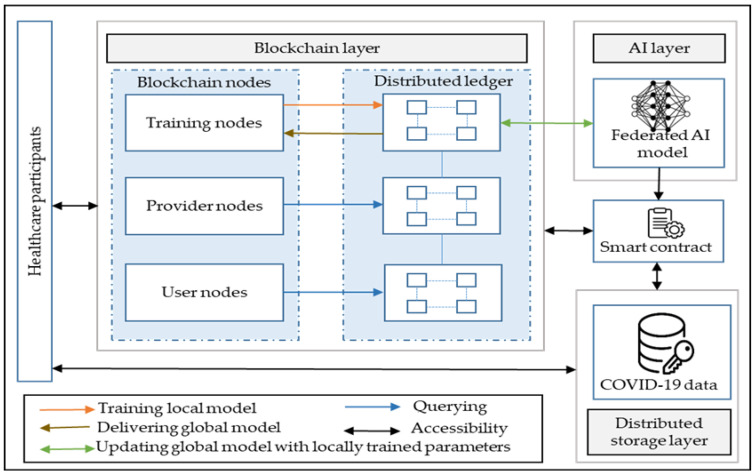
Schematic representation of the envisioned patient-centric framework using blockchain and AI technologies.

In this section, we present a patient-centric digital healthcare framework by integrating AI and blockchain technology. Figure 3 illustrates the schematic representation of the envisioned patient-centric framework using blockchain and AI for COVID-19. The framework is conceptually organized into three layers: blockchain, AI, and decentralized storage layers. These three layers are integrated with the smart contract to make decisions and maintain accessibility within the patient-centric healthcare ecosystem. The blockchain as a decentralized technology enables multiple healthcare participants, such as regulators, researchers, providers, pharma, payers, and government, to benefit from the patient-centric healthcare services and applications.

The decentralized storage layer consists of the P2P storage system. All the coronavirus data from hospitals, clinical labs, patient-generated data (IoT sensors and mobile operators), and several other sources are combined to construct a primary dataset that subsequently leads to big data. These big data are encrypted and stored in a decentralized storage system by utilizing privacy and security features [67,68]. In a decentralized storage system, the data are distributed into different chunks and stored inside various nodes of a P2P network, instead of storing all the coronavirus-related data in a centralized server. The advantages of utilizing a decentralized storage system include security, privacy, no single point of failure, and cost effectiveness [69]. There have been a few successful distributed file-sharing systems, like IPFS, storj, swarm, orbitDB, GUN, skeps, and sia [69,70]. These file-sharing systems combined blockchain technology to enable off-chain and on-chain storage mechanisms. In off-chain storage, data are not publicly accessible, and the transaction agreement happens outside of the blockchain. An on-chain storage mechanism refers to blockchain transactions that are valid when transacted on the publicly distributed ledger. The significant purpose of a decentralized storage system is to enable the distributed and immutable off-chain and on-chain storage networks to facilitate patient-centric management for coronavirus data. For instance, an infected X-ray image has been encrypted for privacy purposes by a doctor and uploaded to the IPFS network to store the image data off-chain. The stored encrypted image returns an IPFS hash value, and this hash value is stored on-chain in the blockchain ledger after being verified by the key participants of the healthcare ecosystem [70]. Key participants can be a doctor, clinicians, and hospital administrators. Thus, combining on-chain and off-chain data storage mechanisms allows the building of a permanently addressable decentralized storage system that could be connected securely to other crucial databases or systems in the world to form a global healthcare network [16,71]. 

The blockchain layer consists of provider nodes, user nodes, and training nodes. The provider node includes participants, such as hospitals, clinics, or healthcare organizations, to store and update every patient information (name, patient’s unique ID, prescribed medicines, and discharge summaries) in the blockchain ledger. Furthermore, provider nodes assign ownership to the patient medical data in the on-chain blockchain distributed ledger, as well as store coronavirus-related electronic health records, such as CT scans, chest X-rays, and medical reports, in off-chain decentralized storage networks. The user node consists of patients who manage and control their coronavirus-related datasets on blockchain platforms. This operation can be achieved by implementing an Ethereum-based smart contract protocol, such as patient-centric access control (PCAC-SC), to enable a distributed and trustworthy access control policy [16]. The smart contract ensures access to the control and safety of patient-sensitive data without using a centralized infrastructure. The participants in the patient-centric healthcare ecosystem are synchronized with the provider blockchain network to share communications between them regarding accessing the patient data for establishing pandemic management and response strategies. The patient-centric approach allows patients to protect and give access to COVID-19 data based upon the healthcare entities’ requests. The blockchain-based, patient-centric framework could offer a number of feasible solutions for coronavirus-related services and applications with improved interoperability among different healthcare platforms, such as insurance claims, pharmaceutical needs, hospital databases, supply chains, and clinical data management. 

The AI layer is integrated with blockchain and a decentralized storage network using the smart contract protocol. The AI layer consists of federated machine learning and deep learning models, where data providers, such as hospitals or clinics, train an AI model locally using the private data obtained from patients and upload only the locally trained AI model parameters to the decentralized storage network. The reference to the parameters of the locally trained AI model is stored in the distributed blockchain ledger to update the global model. For example, let us consider a scenario where two hospitals and one research institute teamed up to build an AI model that can automatically analyze CT scan data for detecting COVID-19 infections. The team employs a blockchain-based federated learning approach to maintain the global deep neural network. Each hospital would receive a copy of the AI model to train the model with a CT scan dataset available in their healthcare infrastructure. Once the AI model has been trained locally in the hospital for a couple of iterations, the participants would send only their updated version of the AI model back to the blockchain network. The contributions from all participants would then be aggregated from the decentralized storage network. The updated AI model parameters are shared with participating healthcare organizations, such as hospitals, to continue the local training. Thus, hospitals only share weights and gradients by keeping their patients’ sensitive data privately within their healthcare infrastructure. Here, blockchain technology distributes the AI model parameters among hospitals. The decentralized architecture for hospitals can share their data among multiple healthcare organizations without any leakage of the patients’ privacy. The smart contract in the framework ensures a decentralized trust among the involved participants by defining rules for the model training agreement and automatically enforcing those obligations [72]. Smart contracts record agreements as a computer code with certain rules. When the rules are satisfied, the agreement is enabled. Smart contracts not only facilitate rule-based accessibility, but also provide flexibility to implement custom federated learning solutions. In addition, smart contracts enable different incentives based on participants’ contributions, restrict operations, and define new rules consisting of upcoming requirements. The trained model in the blockchain network provides better and more accurate predictions, because it holds the most up-to-date information about COVID-19 symptoms. These models are deployed to disseminate and analyze data, which can directly impact patients, service providers, and other participants of the patient-centric healthcare ecosystem. Furthermore, blockchain can accelerate the development of data-hungry AI applications using rule-based smart contract protocols [73], since, in healthcare, patients’ archival data need to be immutable and accessible only to specific researchers for privacy purposes. 

The use of smart contracts for rule-based model training is a novel concept, and blockchain is an ideal platform for standardizing health data structures for AI training, clinical trials, and regulatory purposes. The summary of various technical aspects and their benefits for implementing the conceptually proposed framework over traditional healthcare systems are presented in Table 2. The proposed blockchain-based, patient-centric healthcare system could facilitate AI models and large datasets to be widely shared, updated, and trained to increase the rate of AI adoption and effectiveness. AI implication procedures were developed in the healthcare industry for fighting the COVID-19 pandemic by performing accurate analysis and reliable predictions on vast data collected from coronavirus sources. The blockchain and AI technology that supports patient-centered care has to coordinate the flows of COVID-19 data that are coming from a variety of sources through services and applications, such as outbreak estimation, coronavirus detection, drug/vaccine development, coronavirus analytics, future case projections, and performing automated surveillance.

## 5. Applications for Healthcare Management and Response Strategies during COVID-19

**Figure 4:**
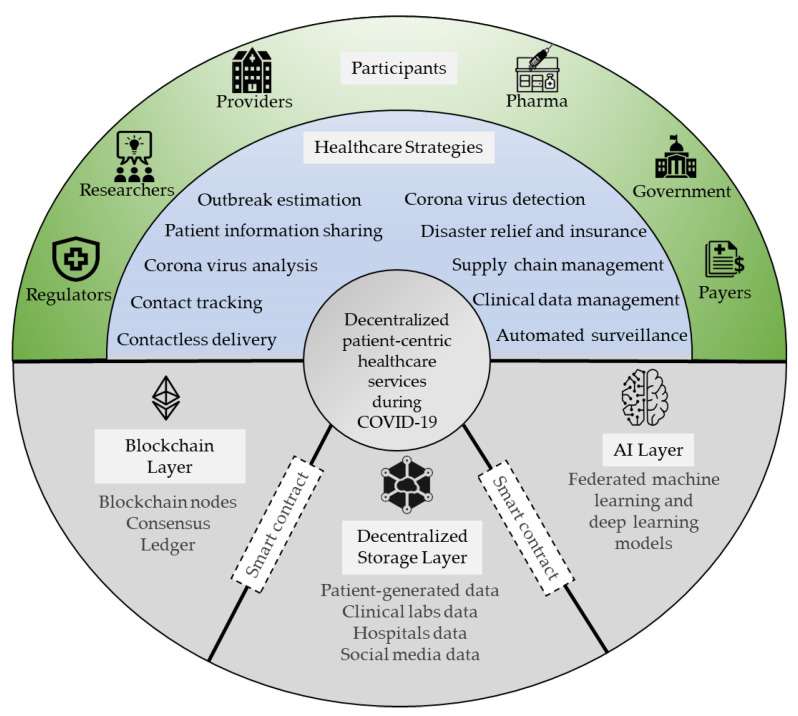
The convergence of blockchain and AI for a decentralized, patient-centric healthcare system to tackle COVID-19.

In this section, we explore the digital service opportunities and applications of the blockchain- and AI-based, patient-centric framework for facilitating COVID-19 healthcare strategies. Figure 4 shows the possible decentralized digital healthcare services and applications offered by the converged blockchain and AI technologies to participants in a patient-centric healthcare ecosystem. The traditional healthcare system has drawbacks, such as limited access to COVID-19 data, participants’ struggles to manage data, and high costs. The proposed patient-centric framework ensures distributed data access and secure logging of digital transactions and at the same time maintains the security and privacy of patients’ data.

### 5.1. Patient-Centric Information Sharing and Clinical Data Management

In COVID-19 research, interoperability among healthcare participants is of predominant importance. However, interoperability mechanisms must avoid violating international and national data-sharing regulations, such as the General Data Protection Regulation (GDPR) compliance and Health Insurance Portability and Accountability Act (HIPAA) [74,75]. These compliances demand that patients be the holder of their data ownership and not organizations, such as hospitals, clinics, or research centers, that generate or create the revenue from data. Therefore, it is necessary to enable individuals to control their COVID-19 data for better communication between healthcare organizations and caregivers to obtain a higher standard of care. The patient’s COVID personal health information (PHI), such as scanned medical images, blood oxygen level, heart rates, medication doses, and history of health conditions, are gathered with high privacy through medical IoT (MIoT) and AI-powered decentralized applications (dApps) developed on the blockchain using smart contracts. These gathered data are uploaded directly to the proposed blockchain-based system to eliminate data forging and mutation issues. In addition, the proposed decentralized framework enables patients and physicians to easily participate in telemedicine rather than visiting hospitals during the pandemic. The clinical data are managed using distributed ledger technology and P2P networking features with no centralized management costs, apart from the minimal fees of the Ethereum network [9], thus enhancing patient empowerment.

In general, the actual COVID-19 data are not stored in the blockchain; instead, the metadata of the COVID-19 health information is encrypted and maintained in the blockchain as a pointer. The actual information collected from data sources is stored off-chain in a decentralized storage network, such as IPFS, swarm, and sia. Thus, the blockchain offers security and privacy by storing patients’ personal health information in a decentralized storage network. There are several techniques and prototypes that have been recently adopted by researchers to implement permissioned blockchain-based patient information sharing, such as MedChain [76] and MedRec [9]. These healthcare prototypes provide the patient with full control over his or her medical records and enable data sharing and authentication processes. Incorporating such techniques in our proposed architecture eliminates the costly middleman who manages the centralized databases and enables patients to have full control over their data. In addition, a decentralized, patient-centric approach allows data monetization through smart contracts, such as patients retaining their data ownership and remunerating with tokens for their sharing of COVID-19 data with participants, such as researchers. For example, Health Wizz [77] uses blockchain to tokenize data to enable patients to securely aggregate, share, donate, trade, and organize their PHR. Thus, a patient could monetize their COVID data for training an AI model for faster and accurate diagnosis using the patient’s X-Ray or CT scans or for predicting the future outbreak [63]. The patient-centric approach helps to protect patients’ privacy and maintains trust among participants by providing transparency in storing and sharing data. Smart contracts in the blockchain can serve as security promoters for clinical trial data [78].

### 5.2. Decentralized Contact Tracing

Contact-tracing applications are among the key digital healthcare services helping to fight the COVID-19 pandemic. There are several technologies that could provide contact tracings, such as mobile phone platforms like quick response (QR) codes, Bluetooth, mobile applications, social graphs, contact details, network-based API, GPS, and wi-fi. However, data privacy and security concerns remain as hurdles that could complicate the process of identifying virus-exposed persons [79]. The decentralized, patient-centric approach based on blockchain and AI can ease those concerns by balancing public health needs with privacy concerns and data analysis [80]. Individuals use blockchain technology to securely share their personal information without revealing their identity to public health agencies, such as a government-aided corporate database or government health authorities. This could help to notify individuals who come into contact with the coronavirus-infected patients without sharing the other personal or medical data of the infected persons [81]. In addition, the gathered data can be used to identify the clusters and hotspots by analyzing the data through an AI model. The outcome of the AI model could be used as a key tool to facilitates a more responsible reopening of the economy without causing a surge in the case. Researchers utilize blockchain technology to issue blockchain-protected digital COVID-19 vaccine passports to immunized citizens. These health certificates can be authenticated easily by public health authorities to verify the status of an individual [82]. 

### 5.3. Outbreak Estimation

The general coronavirus data, such as the number of newly infected cases, death cases, recovered cases, and infected regions, are obtained from media platforms to estimate the risks of the infection and its likely spread using AI models. AI enables the identification of the most vulnerable people and countries, and predicts the number of positive cases to take measures accordingly in advance. In addition, AI can combat COVID-19’s spread by analyzing people’s phone usage patterns to estimate the outbreak size, considering the fact that a COVID-infected patient or a dead person’s phone usage patent will change, since the phone might be used by a family member or will be idle. These pattern changes could be detected by analyzing the datasets obtained from wireless operators using AI models [83]. Sarker et al. [84] used ML to model and predict the phone usage records of individuals through learning their personalized diverse mobile activities. Furthermore, J. Shen et al. [85] used DL to accurately estimate the mobile phone application usage, i.e., abnormal calling behaviors and phone service inactivity. Specifically, deploying blockchain-enabled dApps on patients’ mobile devices helps to forecast and track the spread of the virus infection globally by anonymously preserving the patient information.

### 5.4. Coronavirus Detection and Analysis

AI facilitates an automated decision-making system, which helps to develop a new cost-effective diagnosis system for the COVID-19 cases using ML and DL algorithms. For instance, facial recognition [86] using AI helps to detect the temperature on a human face and predict whether the person is wearing a mask or not so that healthcare officials can identify the COVID symptoms and violations related to COVID-19. AI enables computerized diagnosis of the infected cases by analyzing CT, chest X-rays, and MRI scan images. The authors of [87] proposed a detection system using two-dimensional and three-dimensional DL algorithms that were combined with available AI models to identify COVID-19 thoracic CT features with high accuracy. The work in [88] proposed a CT image analysis model to screen COVID-19 by differentiating COVID-19 pneumonia characteristics from influenza A viral pneumonia. The authors used a pulmonary CT image set for feature detection and to differentiate influenza A viral pneumonia, COVID-19 viral pneumonia, and healthy cases. In another study [89], convolutional neural network-based models were used to analyze chest X-ray radiographs to detect patients’ viral pneumonia caused by the COVID-19 virus. From the aforementioned examples, we can note that coronavirus data plays a crucial role. However, the healthcare data for training the AI models are fragmented and stored in a private server to maintain privacy. Thus, creating a robust result across populations is a difficult task. The decentralized, patient-centric framework facilitates a federated learning approach to train a shared AI global model within a blockchain network. Meanwhile, keeping all the sensitive patient COVID-19 data in the decentralized storage network, ensures to connect the fragmented healthcare data sources with privacy preservation. This approach increases trust by allowing advanced machine learning to be developed on distributed data with full respect of the confidentiality for the data providers and maintains the property rights of the companies that propose the machine learning models.

### 5.5. Disaster Relief and Insurance

COVID pandemic situations are often accompanied by significant threats, such as disrupting the healthcare delivery infrastructure and extending the effect of the physical health of individuals living in affected communities for short- and long-term periods due to nationwide lockdowns and social distancing rules. Governments, financial organizations, and healthcare delivery infrastructures are compromised by the loss of facilities; difficulties in providing loans and other financial lifelines; scarcity of health professionals in the impacted area; and disruption of critical supports, such as information sharing and data management technology, pharmaceuticals supplies, supply chain management, and medically necessary social services. The key reasons for such challenges are that the existing healthcare infrastructure is centralized, non-transparent, and relayed on paper-based procedures, which consumes more time and is ineffective.

The blockchain smart contract in the decentralized, patient-centric approach eliminates third-party intermediaries and the inherent processing delays associated with traditional paper-based policies. The smart contract simplifies complicated applications to ease the approval process for providing insurance and loans with reduced operational risk. The policy agreements are created and deployed in blockchain networks by the participants, such as governments, pharma, and regulators, to enable fast, reliable, and scalable solutions in the patient-centric healthcare consortium. Furthermore, patients are now at the center of healthcare operations, such as the revenue cycle [90]. Therefore, patients have choices for the providers they utilize, such as the hospitals, payers, and pharma, and the providers are now expected to deliver retail-like service levels. This patient-centric healthcare model calls for new solutions in infrastructure, technology, and mindset to ensure a transparent financial experience for both the patient and provider. Another application includes managing the medical billing data by AI. The AI model helps to learn about health plans by feeding the sensitive databases into the AI models from different platforms stored on a blockchain. AI would analyze the necessary sensitive information in the blockchain provided by the appropriate entity, disseminate the understandable data, and provide answers related to health plans and medical bills.

### 5.6. Supply Chain Management

The healthcare provider and the supply chain play a critical role in protecting the patients’ safety and treatment. The shockwave of the COVID-19 pandemic created disruptions in the global supply chain due to the abrupt changes in delivery routes, individuals buying patterns, and supply shortages. For example, there was a surge in demand for household essentials due to panic buying. Specifically, COVID-19 exposed the difficulties in managing medical equipment and pharmaceutical supply chains due to the lack of integration and alignments of interest in the healthcare supply chain. Therefore, it is necessary to critically redesign the existing supply chain system to manage the flow of medical supplies and respond to additional challenges presented due to the ongoing global pandemic.

The decentralized, patient-centric approach plays a crucial role in designing a more resilient, fully connected, and trustworthy supply chain environment using blockchain technology. Here, the blockchain anonymously considers various stakeholders with uniting factors, such as delivering a higher quality of care and improved customer service. The patients will be at the center of the healthcare supply chain and integrated with the system data using blockchain-enabled dApps. Thus, immutable recording of data logs supports auditability, transparency, and provenance. By using a blockchain, supply chain organizations achieve a rapid flow of supplies from the origins to the destinations in a reliable and trusted manner. These help to harmonize just-in-time manufacturing with disaster preparedness. Meanwhile, a well-programmed smart contract gives a high level of automation and access restrictions to save billions of dollars and thousands of lives [91]. The patient-centric supply chain management system utilizes AI to increase the system’s efficiency for reporting and analyzing the obtained data from medical and logistic teams. The supply chain data are interpreted to derive insights that will enable continued tweaking of the system for even better results.

### 5.7. Contactless Delivery and Automated Surveillances

Contactless delivery eliminates direct communications among people (e.g., person–person interactions changed to person–machine or person–machine–person) [92] and delivers healthcare services or products between individuals, patients, healthcare professionals, and other providers through digital technologies. Thus, many people and mildly infected patients prefer contactless online treatments and contactless (automated robotic) delivery of essential supplies, such as medicine and food, during a lockdown or quarantine period. The goal is to maintain an individual’s safety by avoiding face-to-face interactions and sending a person for doorstep delivery. Accordingly, telemedicine services were naturally contactless and widely practiced in the pre-COVID period. The telemedicine services encompass remote consultation through audio/video calls using a robot with a camera and video features with a tablet, smartphone, or computer. In telemedicine services, patient data are generally stored and managed through a centralized platform [36]. Hence, there is a possibility of security attack, raising questions regarding the patient’s privacy of data. In addition, it is necessary to monitor people who violate COVID-19 rules by not wearing facemasks and not maintaining social distancing. 

To address challenges, drones and robots are used for contactless delivery and automated surveillance in response to COVID-19, such as monitoring public space; providing guidance during lockdown and quarantine; lab sample pick-up and delivery; transporting medical supplies to minimize the transportation times and infection exposure; and aerial spraying of public areas in order to disinfect potentially contaminated places [29]. Though the utilization of AI technologies facilitates UAVs and robots to precisely operate and execute the task without human interventions, it leads to security attacks, such as device hacking, data theft, and modifications in robot functions. A blockchain- and AI-enabled, patient-centric approach offers a vast range of possibilities to mitigate the issues by integrating with technologies, such as robots and UAVs. The blockchain enables independent drones or robots to reach a consensus in a decentralized way, and shares knowledge to improve the performance of the system [93]. The blockchain, along with smart contracts designed to ensure a high level of security, automates the operations of robots [94] under policies implemented via the government or healthcare organizations’ supervision. Blockchain and AI empower personalized care for patients and monitors people using secured and automated UAVs and robots.

## 6. Discussions

### 6.1. Challenges and Solutions

Healthcare professionals and medical industries around the globe are urged to fight the pandemic with rapid screening, forecasting, contact tracing, and the development of drugs or vaccines with more accurate and reliable operation. Blockchain- and AI-based, patient-centric approaches enable a personalized healthcare service to patients and healthy people. However, some challenges must be addressed for a patient-centric approach to embrace blockchain and AI technology and to leverage maximum benefit.

The first challenge is related to the increased volume of raw clinical data, and the verification of new transactions can take time on the blockchain, depending on the consensus algorithm. For example, the latency for PoW is higher due to the time taken to approve each transaction by the blockchain infrastructure, which leverages scalability issues. The scalability issues could be overcome by utilizing a permissioned blockchain built to handle large transaction volumes without time-intensive validation. Recently, researchers have been developing novel solutions, such as sharding [95], to achieve network-wide scalability by dividing rapidly growing blockchain networks into groups called shards. Furthermore, designing specific hierarchical blockchain systems and consensus algorithms could help to resolve the scalability issues. 

The second challenge is related to privacy and security issues. The blockchain data are distributed to all the nodes which, in turn, leads to non-compliance with privacy laws (e.g., HIPPA and GDPR) and vulnerabilities [96]. Therefore, it is necessary to store data off-chain in order to maintain data privacy and security. The privacy of data can possibly be achieved by new privacy methods, such as homomorphic and attribute-based encryption, secure multiparty computation, zero-knowledge proof, obfuscation, and format-preserving encryption. The different security levels in a system could be accelerated by designing with hybrid privacy methods and using security-enhancing technologies, such as a homomorphic signature [97], which works better than public key certificates. More importantly, COVID-19 data gathered from hospitals, clinical labs, and patients can be altered by any malicious attacker and makes AI learning invalid. Therefore, it is necessary to collect the COVID-19 data without any privacy leakage from different sources using federated learning combined with blockchain technology. Possibly the largest barrier to the adoption of a patient-centric framework based on AI and blockchain relates to legal disputes or regulatory issues. The central entity of each healthcare organization is liable for any legal issues and is responsible for the overall smooth functioning of the centralized healthcare systems. However, a decentralized, patient-centric system leads to difficulties in solving any legal dispute or discrepancies in the public blockchain infrastructure. For example, copyright infringement and defamation problems arise when personal information runs on converged AI and blockchain platforms. In addition, countries are reluctant to share coronavirus-related databases, which creates additional difficulties in performing a large-scale AI operation. Therefore, regulatory approaches would need to be cleverly balanced by developing corresponding administrative processes and a new legal framework among countries and healthcare organizations, such as the WHO, while recognizing the possibility of the technology [98]. 

### 6.2. Future Work

In short, the proposed conceptual framework tries to generalize the process of a decentralized, patient-centric healthcare ecosystem to fight the COVID-19 situation with a clearer understanding of possible applications and functionalities. The framework takes full advantage of blockchain and AI technologies to establish better solutions in solving pandemic-related issues. In our future work, we aim to implement the conceptually proposed framework and test our system with the applications related to healthcare strategies. We will focus on conducting experiments by optimizing the blockchain technology to achieve better performance in terms of improved security and increased throughput. In addition, we will deploy a real-time adaptive AI architecture model using a blockchain-based federated learning approach with the potential to manage multimedia healthcare data for predictive modeling, patient monitoring, and emergency department operations in response to critical healthcare situations. Democratizing aspects of healthcare provide personalized care, as well as save time and money for patients. Moreover, this conceptual framework will motivate researchers to pay more attention and explore the combination of other technologies, such as drones, smart MIoT, robots, and digital twin technologies, to help fight future epidemics and pandemics.

## 7. Conclusions

In this paper, we provided an overview of digital healthcare services in response to COVID-19 pandemic management services. Then, we presented a conceptual framework for a decentralized, patient-centric healthcare system by integrating blockchain and AI technologies to fight against the coronavirus epidemic. The proposed decentralized, patient-centric framework can contribute in four ways. Firstly, it improves the interoperability of different healthcare platform stakeholders, such as providers, payers, pharma, governments, and researchers. Secondly, patients store COVID-19 health records securely on the patient-centric blockchain platforms and own their sensitive data. Thirdly, the blockchain reduces siloed patient datasets and eliminates centralized organizations, thus improving medical research and treatment by using AI models for predictive diagnosis and analysis. Fourthly, the blockchain enables federated learning techniques, where AI models train the COVID-19 data with the patients’ permission at the hospital side while preserving their privacy, and aggregates the knowledge from the nodes to learn a global model. Here, only the global AI model parameters are shared with the hospitals, and once the training is performed locally at hospitals, the model parameters are sent back for aggregation. Thus, the machine learning or deep learning models are trained collaboratively in the distributed network while maintaining hospital and patient data privacy. In addition, we explored the possibilities and potential applications of these combined technologies to facilitate the traditional public health strategies for tackling COVID-19, such as contact tracing, outbreak estimation, coronavirus detection, analysis, clinical data management, supply chain management, contactless delivery, automated surveillance, disaster relief, and insurance. The acceptance of a patient-centric healthcare model could transform the centralized healthcare system into a decentralized healthcare system, thus placing the patients at the center of the healthcare ecosystem to control, access, and share their healthcare data, facilitating research and personalized treatment.

## Figures and Tables

**Table 1:** Summary of the COVID-19 pandemic’s management and response strategies [40,41].

Strategies	Functions	Digital Technologies	Challenges
Contact Tracking	Identifies and monitors individuals that come into contact with an infected person within a specific duration of time.	Bluetooth Low Energy technology, mobile phone applications, wearables, and IoT devices.	Security and privacy issues, since individuals’ data are analyzed and stored in a centralized cloud system.
Quarantine and Self-Isolation	In quarantine, individuals are requested to stay in a place (i.e., home or government facilities) for 14 days after being exposed to a COVID-19-infected person. In self-isolation, an infected person isolates within a house or other location to prevent contacting uninfected persons.	AI, a global positioning system, cameras, and recorders.	Breaches civil liberties, restricted access to essential services, and fails to track the individual who runs away from a quarantine facility without their device, like a mobile phone.
Automated Surveillance	To identify and monitor individuals without facemasks, social distancing, and accidental touching in public gathering places. Detects symptoms, such as breathing difficulties, coughing, and fever, using self-tracking digital technologies.	Facial recognition, digital thermometers, surveillance cameras, and thermal cameras.	Security attacks, operational cost.
Clinical Data Management	Used to diagnose infected individuals and provides the capacity for telemedicine services and virtual care, prediction of clinical outcomes, and monitoring of clinical status by clinicians.	Picture archiving and communications system (PACS).	Not cost efficient, privacy breaches may occur, failure in diagnosis.
Patient Information Sharing	Patient health and medical information sharing could decrease the possibility of duplicate testing and avoid medication errors. Furthermore, sharing patient data among the global research community plays an essential role in coronavirus research by formulating powerful raw data sets.	AI, web-based toolkits, and PACS.	Satisfying Health Insurance Portability and Accountability Act (HIPAA) compliances, lack of anonymity, security, privacy, and data management issues.
Contactless Delivery	During the lockdown, contactless delivery of essential supplies, such as medicine, food, and sanitizers, prevents direct interactions with people, since doorstep delivery might not be safe during a high transmission rate.	Robots and drones.	Security attacks, operational costs, and legal issues in the case of an accident.
Supply Chain Management	Identify and secure logistics capacity based on the type of goods, such as medical equipment and vaccines/drugs or other pharmaceutical medicines.	Mobile platforms, data analytics, cloud, and IoT.	Procuring medical equipment, pharmaceutical medicines, and household essentials are difficult due to the surge in demand.
Disaster Relief and Insurance	Financial organizations and governments have to help the public by providing unemployment insurance relief, loans to protect their business losses, and health insurance that covers treatment costs during the COVID-19 outbreak.	Web-based toolkits and mobile applications.	Time-consuming and ineffective due to paper-based procedures and centralized authority.

**Table 2:** Comparison between traditional healthcare systems over proposed healthcare system based on various technical aspects and their benefits.

Aspects	Standard Healthcare System	Proposed Healthcare Platform
Source Data Storage	The COVID-19 data are stored in a centralized cloud-based storage system, like PACS.	The COVID-19 data are stored in decentralized storage systems, such as IPFS.
Database Sharing Mechanism and Integrity	Depends on a cloud-based mechanism and EHR databases managed by a third-party clearinghouse. Thus, there are possibilities of data tampering.	Depends on a blockchain-based sharing mechanism and EHR databases managed by the participants of the healthcare ecosystem. Thus, databases are immutable.
Administration Performance and Scalability	More transactions are processed per second and enable great scalability.	Process minimal transactions per second, and there are scalability issues since the framework is at its developing stage.
Implementation Cost	Easy to implement and maintain due to its large-scale adoption.	Uncertainty in the operating costs.
Incentive Mechanism for Sharing Data	Not available.	The patient can receive an incentive for sharing their medical data for research purposes.
Data Accessibility	Depend on healthcare entities.	Patients have complete access to and control over their data.
Anonymity	High risk of privacy leakage and identity theft.	The identity of the patients and the transactions between healthcare participants remain anonymous since blockchain public addresses do not link to anyone’s identity.
Data Auditability	Always depends on administrators to audit the data.	The moment the blockchain reaches a predetermined state, any node in the blockchain network can track and trace the data right from its origin based on cryptography technology.
Computational Performance of AI	Computationally expensive for training large datasets acquired from different sources in a centralized server.	The federated learning approach reduces the computational power by enabling collaborations between several healthcare organizations to train the distributed global AI models without relying on any centralized server.
Decision Making	Human involvement.	Human involvement, AI, and a smart contract.
Fault Tolerance	Risk of a single point of failure.	A distributed blockchain ledger is highly fault-tolerant because of the consensus mechanism.

## Data Availability

Not applicable.
